# Some Statistic Studies of the Cures of Inebriety

**Published:** 1911-12

**Authors:** T. D. Crothers

**Affiliations:** Superintendent Walnut Lodge Hospital; Hartford, Conn.


### Some Statistic Studies of the Cures of Inebriety

By T. D. CROTHERS, M D.
Superintendent Walnut Lodge Hospital
Hartford, Conn.

A HALF a century has passed since the question of the cure of inebriety by medical means was first discussed by physicians. Some of the early studies were very curious groupings of figures and statements. Others were startling results claimed to have followed the application of certain remedies, but in all there was a timidity and assertive dogmatism common to all early studies of new subjects.

The empirics and persons with limited experience, have claimed a percentage of cures by the application of medical means, that ranged all the way from 60 to 90 per cent. The practical scientific workers while asserting that many cases have been positively cured, have hesitated about the percentage and the extent of the curability. This general experience continues up to the present.

Much of the literature that is reliable on the causes and treatment of inebriety has turned on the individual conception and theories of what inebriety is and is not. Many persons believe that inebriety is simply an abnormal thirst, or craze for spirits, and when this is controlled, the patient is cured.

They reason that the desire for spirits is a mental condition which can be reached by will power, and its reappearance is largely under the control of the patient. Persons holding such theories report a high degree of curability.

Empirics who claim to have discovered some new remedies, assert that all inebriates are curable. Even the Three Days' Cure promoters are emphatic in their claims that the drugs they give, permanently destroy the drink symptoms. Others more conservative, believing that inebriety is a half-vice and half-disease are confident, that if the methods they described are used, at least half of all patients will be cured.

Many good physicians belong to this class, and when asked for data on which they rest these conclusions, it is found to be based on the histories of patients, extending one or two years back. They reason that if the patient abstains from spirits for this time, the disease is permanently eradicated.

Many persons believe that inebriety is purely a moral condition, to be treated by moral means, such as prayer and the pledge. They claim a very large proportion of all persons who are sincere in following the means which they present will be cured. Thus temperance reformers, evangelists and church people, as well as empirics, present a startling statistic array of cures, and the impression is created, that if these were real and veritable, inebriety would soon disappear, and be stamped out.

Hospitals and sanatoriums where these cases are treated with some scientific precision, seldom publish exact data of cures, but use the term "recovery" as describing the present condition of the patient, or that which has followed after a short interval. Tables of results show a certain number of persons who have recovered, and others who have failed.

The recovered persons frequently remain in this condition for years, but the approximate number of these is not certain.

Practical studies of these persons by trained physicians show that the drink craze is only a symptom and not the disease, and this symptom of course, is easily controlled. A great variety of means and combinations of drugs will break up this drink symptom for a time, but its return is dependent on other causes which are not reached by the so-called specifics and drugs, used to cover up the symptoms.

The real causes are complex and very largely depend on the individual and a variety of conditions. The demand for drink is simply the call for relief from some physical and psychic depression and irritation, local or general. Spirits afford an anaesthesia which covers up this irritation and psychic fatigue.

The nervousness including excitement, depression and a great variety of other conditions, which are suppressed by spirits, breaks out again, and the means which produced relief at one time, are called for again.

Unlike many other drugs, the anaesthesia of spirits creates a demand for its repeated use, and this becomes a veritable obsession that is both psychic and physical. This condition is often inherited in low vitality, feeble resisting power, and a constant effort to overcome it, is a part of the disease. It is acquired by neglects, strains and drains, and everything which disturbs the normal relation of body and mind.

No other substance known, has such peculiar anaesthetic action on the motor and sensory cells. A certain periodicity is created, in which this anaesthetic relief is called for, with great intensity. This particular obsession for relief from alcohol is self-limited in many cases, and after a time dies out.

The degeneration which the use of alcohol has formed remains, but the drink craze disappears. What pathologic changes have occured in the organism, whether produced by other drugs or remedial forces to bring this about, is unknown.

The subsidence of the drink craze is often considered as due to the specific action of certain drugs or certain particular methods of treatment. The return of the drink symptom and the failure of these particular means show that the former deduction was wrong.

Innumerable cases are noted of the sudden subsidence of the drink craze both with and without any special treatment, and considerable literature on this subject is devoted to theories, how and why this occurs. Leading authorities in this field, recognize the physiologic change, and seek to encourage it by the use of constitutional means and measures, to build up and restore the organism to its previous normal condition.

In the effort to determine the curability of the average inebriate, it is essential to know the general causes and conditions, both inherited and acquired. This forms a basis for the probable permanency of the subsidence of the drink craze, and the restoration of the patient. Some of the studies based on these general considerations, began first at Binghamton in 1874. The New York State Inebriate Asylum at that place, in reply to the criticisms concerning its work, made a statistic study of 1,200 patients, who had been under treatment six years before the inquiry was made.

The friends, relatives, and patients themselves, were written to, concerning the present condition and general health of the patient. There were over 900 replies, showing that 62 per cent. of all persons living, were still temperate and restored.

These data were criticized and the late Dr. Willard Parker, the President of the institution, ordered a re-examination of the returns, and further correspondence with the patients. The old data were confirmed and it was shown that over 60 per cent. of patients who had been under treatment five years before, were living temperate normal lives.

Three years later, in 1879, the late Dr. Joseph Parrish of Philadelphia made a study of the history of 100 persons who had been under treatment for inebriety ten years before. Sixty-one persons responded to the inquiry and 39 per cent. were restored and practically cured. These data were studied again and fully confirmed.

The late Dr. Norman Kerr of London, England, made a collective study in 1884 of the histories of persons who had been treated medically for inebriety in sanatoriums and in their homes by physicians. His data showed that six years after treatment 40 per cent. were total abstainers and well. The most thorough study of the curability of inebriety was made by Dr. L. D. Mason of Brooklyn, N. Y. in 1889, of patients who had been under treatment at Fort Hamilton Hospital for inebriates.

Of 4,600 patients who had been treated, from six to ten years before this time, 41 per cent. were temperate and cured. A large number had disappeared, died, and become insane. Many were lost sight of, but the records showed that this large number had received permanent benefit.

The late Dr. Albert Day of the Washington Home, Boston, Mass., attempted the history of the inmates of that home, particularly those who had been away over ten years. The result of several thousand circulars sent to the friends of the patients, succeeded in getting the history of over 40 per cent. Of this number, 20 per cent. were reported as cured and restored.

These were approximate figures and as the inmates of this institution were largely of the chronic class, and their treatment had been brief, the facts were very encouraging.

In a personal experience of thirty-five years, covering several thousand cases, I have repeatedly made studies of small groups of inebriates under treatment for from six to twelve years before the inquiry was made. The general results have shown that one out of every three is permanently restored, or in other words is still temperate and living a normal life after that interval of time.

These and many other studies made from a small number of cases, indicate that about 30 per cent. of all persons who are treated from three to six months, in well organized institutions, remain restored and can practically be called cured.

There are great difficulties in the effort to determine the exact results of treatment in such cases. Thus persons who have been under treatment in an institution, through pride and other motives, conceal all evidence of such treatment, never referring to the hospital, and regarding it as a shadow in their past history. These are the practically cured patients.

On the other hand, persons who relapse are most vociferous in reporting their previous treatment and the failures of the institution with general criticisms of the methods. In this way the incurables are very easily studied, and those who are restored are very difficult to trace.

Experience shows that egoistic supporters of quack and specific cures, and those who boast of permanent restoration are incurable as a rule, and relapse, sooner or later. Hence, certificates of cures are very unreliable, and efforts to build up an institution, on the endorsement of its inmates are unsatisfactory.

A certain number of persons, after treatment in an institution, remain for many years restored, then suddenly relapse and die. Others, after years of abstinence, become drug takers or suicidal. Many chronic inebriates, after the subsidence of the drink craze, develop diseases of old age, and become degenerates and die early.

While the drink craze dies out, and the active or contributing cause, alcohol, is removed, defects of the brain and nervous system remain, or develop into some other form of degeneration and disease.

Many observers who manage small hospitals for this class, have determined from their experience, that the claim of from 25 to 30 per cent. of permanent restorations, is verifiable.

The removal of certain specific causes is followed by restoration beyond any question or doubt. Often the value of hospital treatment depends largely on discovering the nature of the causes of inebriety, removing them and training the patient to avoid them in the future.

Patients in all degrees of chronicity are restored, and the possibilities of treatment along practical scientific lines are very great. Some of the public institutions of Great Britain, particularly in the neighborhood of London confirm this in a most practical way.

In one large farm, managed by the London County Hospital, where degenerate and incurable inebriates are received, the degree of curability from farm work, control and uniform living is very pronounced. Experience in private institutions for mental disease confirms this. Many of the inmates of these places are drink and drug victims, poisoned and exhausted, but from change of life, improved nutrition, habits and thoughts in new environments, recover.

There is a great wealth of illustration confirming this in the experience of private hospitals and sanatoriums. Many of the inmates who are called nervous, over-worked men and women are inebriates in the first stages, and their recovery, follows as a rule, particularly if the patients recognize the real and active causes.

Some of the great sanatoriums of the country are supported very largely by persons whose disability is due to the moderate or excessive use of spirits, continuously or at intervals. The curability of such patients is the every day history, and the struggle to make it permanent, brings out a great variety of interesting clinical studies.

On the other hand the Government efforts and the legal measures to treat inebriety in jails and prisons, are terrible failures and actually cultivate and grow the very conditions they seek to prevent.

Eminent clinicians affirm that if alcohol and syphilis could be eliminated, a very large proportion of the diseases of today and the mortality would diminish. Instead of 30 per cent. the evident ratio of curability today, this number could be greatly increased.

It is a very gratifying fact to know that the percentage of cures noted in hospitals for the insane is very largely increased by the results of treatment of the alcoholic patients. During the last ten or fifteen years inebriates in various stages, are sent to the hospitals for the insane for treatment. Wards are set apart for them and their treatment and restoration, constitute a very important part of hospital work.

It is evident from the great variety of facts that inebriety is a curable disease. Ignorance of the subject and confusion of opinions surrounded with empiricism, make it a favorite field for quacks. It should be a field of practical medicine for every physician, and may be treated in home and office as well as sanatoriums.

While sanatorium treatment is undoubtedly the great essential in the change of surroundings, there are many records published of home cures with a high percentage. This of course depends on a great variety of peculiar causes.

Such treatment calls for psychic as well as therapeutic measures, and when used in early stages of the disease, there can be no doubt of their success. Almost every physician with a large practice can refer to persons cured at home. Ordinarily he fails to give the proper credit to the means used, considering it more mental and moral than physical. The time is not far distant when inebriety will be treated at home by the family physician, with as much success as any other disease, and also in sanatoriums, where inebriates particularly need a change of living.

The curability is assured beyond all question, but what is needed now is to lift the subject out of the realm of quackery and place it in the field of scientific medicine.

